# Seroconversion and Abundance of IgG Antibodies against S1-RBD of SARS-CoV-2 and Neutralizing Activity in the Chilean Population

**DOI:** 10.1155/2021/6680337

**Published:** 2021-02-19

**Authors:** R. González-Stegmaier, K. Cereceda, J. L. Briones, C. Beltran-Pávez, A. Oyarzún-Arrau, S. Riquelme-Barrios, C. Selman, F. Yarad, M. Mahave, C. Caglevic, R. Morales, A. Aguirre, F. Valiente-Echeverría, R. Soto-Rifo, H. Marsiglia, R. Gazitua, F. Villarroel-Espindola

**Affiliations:** ^1^Translational Medicine Laboratory, Instituto Oncológico Fundación Arturo López Pérez, Santiago, Chile; ^2^Haematology Department, Instituto Oncológico Fundación Arturo López Pérez, Santiago, Chile; ^3^Laboratory of Molecular and Cellular Virology, Virology Program, Institute of Biomedical Sciences, Faculty of Medicine, Universidad de Chile, Chile; ^4^HIV/AIDS Work Group, Faculty of Medicine, Universidad de Chile, Chile; ^5^Diagnostic Units, Instituto Oncológico Fundación Arturo López Pérez, Santiago, Chile; ^6^Biobank, Instituto Oncológico Fundación Arturo López Pérez, Santiago, Chile; ^7^Medical Oncology Department, Instituto Oncológico Fundación Arturo López Pérez, Santiago, Chile; ^8^Cancer Research Department, Instituto Oncológico Fundación Arturo López Pérez, Santiago, Chile; ^9^Internal Medicine Department, Instituto Oncológico Fundación Arturo López Pérez, Santiago, Chile; ^10^Radiotherapy Department, Instituto Oncológico Fundación Arturo López Pérez, Santiago, Chile

## Abstract

COVID-19 is a pandemic caused by SARS-CoV-2. In Chile, half a million people have been infected and more than 16,000 have died from COVID-19. As part of the clinical trial NCT04384588, we quantified IgG against S1-RBD of SARS-CoV-2 (anti-RBD) in recovered people in Santiago and evaluated their suitability as COVID-19 convalescent plasma donors. ELISA and a luminescent SARS-CoV-2 pseudotype were used for IgG and neutralizing antibody quantification. 72.9% of the convalescent population (468 of 639) showed seroconversion (5-55 *μ*g/mL anti-RBD IgG) and were suitable candidates for plasma donation. Analysis by gender, age, and days after symptom offset did not show significant differences. Neutralizing activity correlated with an increased concentration of anti-RBD IgG (*p* < 0.0001) and showed a high variability between donors. We confirmed that the majority of the Chilean patients have developed anti-SARS-CoV-2 antibodies. The quantification of anti-RBD IgG in convalescent plasma donors is necessary to increase the detection of neutralizing antibodies.

## 1. Introduction

Coronavirus disease 2019 (COVID-19) is caused by severe acute respiratory syndrome coronavirus 2 (SARS-CoV-2). It emerged in December 2019 in Wuhan, China [1, 2], and was subsequently declared a worldwide pandemic by the World Health Organization (WHO) [3]. Currently, Chile has reported half a million infected people and more than 16,000 deaths caused by COVID-19. The government and the private sector have implemented several programs to mitigate this pandemic, including PCR and rapid testing across the country, while some institutions have started trials for convalescent plasma. However, these efforts still do not seem to be enough to control this sanitary emergency.

The measurement of serum antibodies has provided crucial data for understanding key aspects of the infection [4]. It is widely accepted that IgM provides the first line of defense during viral infections, before generating an adaptive response with a high-affinity IgG production, which is important for long-term immunity and immunological memory [5]. In patients infected with SARS-CoV-2, immunoglobulin M (IgM) antibodies are detectable around 7 days postinfection and IgG antibodies usually take two weeks to develop [6–9]. The clinical features of COVID-19 vary widely, ranging from asymptomatic to mild or severe forms with multiorgan dysfunction, and most COVID-19 data were derived from hospitalized patients [8, 10–13].

Accurate quantitative measurement of anti-SARS-CoV-2 antibody responses is essential for public health interventions and therapeutic applications [14, 15]. The SARS-CoV-2 shows two structural proteins as major immunogens, the spike (S) and nucleocapsid (N) proteins, with the RBD domain of the S protein being responsible for the infection of respiratory epithelial cells via interaction with the cell surface receptor angiotensin converting enzyme 2 (ACE2). Antibodies against this segment have been reported as antiviral based on their neutralizing activity in plasma [7, 13, 16–18]. In this study, we quantified the abundance of IgG antibodies against the S1-RBD fragment of SARS-CoV-2 in people who recovered from COVID-19 in Santiago, Chile. After analyzing 639 convalescent participants, 72.9% showed anti-RBD IgG seroconversion with a concentration range in serum between 5 and 55 *μ*g/mL, and no significant correlation with gender, age, and days after symptom offset were observed between groups. Further analysis showed that serum samples with antibodies above 20.9 *μ*g/mL showed a statistically increased proportion of neutralizing antibodies (Nab) against SARS-CoV-2 during convalescence. Regarding early seroconversion, 14 of 26 cases within the first two weeks of severe COVID-19 showed positive levels of anti-RBD IgG with a mean of 21.2 *μ*g/mL, and 7 cases were above the 75th percentile. Our results confirmed a heterogeneous but specific IgG seroconversion after SARS-CoV-2 infection within the population from the metropolitan area of Chile. The production of antibodies against the RBD of spike were shown to be as early as the first 14 days after symptoms started, and those can persist in the blood for almost two months and display protective neutralizing activity during active disease and convalescence.

## 2. Materials and Methods

### 2.1. Patients and Samples

This research consisted of 690 samples distributed in 3 groups: 639 convalescents, 26 active COVID-19 cases, and 25 healthy never-exposed people as a negative control (supplementary Table 1). There were a second cohort of 72 convalescent plasma donors for the analysis of neutralizing antibodies and a third cohort of 100 unrelated cases (positive and negative COVID-19) for ELISA validation. Recovered volunteers and convalescent plasma donors met all criteria for the clinical trial NCT04384588 and signed the specific consent letter. All volunteer patients who recovered from COVID-19 were asymptomatic for at least 21 days and showed a negative result for SARS-CoV-2 by real-time polymerase chain reaction (PCR) test. Volunteers with active COVID-19 consented at the moment of hospitalization and met the following inclusion criteria: (a) patients over 18 years old and (b) COVID-19 diagnosis at enrolment confirmed with a positive SARS-CoV-2 PCR in a nasopharyngeal swab.

All SARS-CoV-2-related samples used in this research were collected between July and August 2020, in Santiago, Chile.

Healthy group samples were considered as plasma from a regular blood donation obtained before October 2019 (never-exposed cases) and as a single blood sample drawn in April 2020 from asymptomatic healthy volunteers with a negative SARS-CoV-2 PCR result (contemporary cases).

For the active cases, treatments given before blood collection are described in supplemental Table 2.

### 2.2. Quantitative IgG ELISA Anti-RBD S1 SARS-CoV-2

Anti-SARS-CoV-2 IgG ELISA test was developed and validated in the Translational Medicine Laboratory of Fundación Arturo López Pérez (Cereceda et al., submitted); a comparative table (Table S3) is included as a supplementary data. Briefly, MaxiSorp™ 96-well microplates (439454, NUNC, Thermo Fisher Scientific) were coated with 50 ng of S1-RBD protein of SARS-CoV-2 (RB.230-30162-100, RayBiotech) in 0.1 M carbonate buffer pH 9 and incubated overnight at 4°C. The coating solution was removed and each well was washed once with cold phosphate-buffered saline solution (PBS). An uncoated surface was blocked for 4 hours at room temperature using 400 *μ*L of blocking solution containing 5% skim milk in PBS pH 7 and supplemented with 0.1% bovine serum albumin (BSA), 0.1% nonimmune donkey serum (017-000-121, Jackson ImmunoResearch), and 0.1% Tween-20. Later, the solution was eliminated and the plates were air dried before storing and freezing at -20°C. Frozen plates were shown to be stable for up to 45 days based on the measurement of the interwell variation coefficient of blanks and positive controls. Before testing the unknown samples, the plates were thawed at room temperature and the excess blocking solution was removed using a washing solution (0.1% Tween-20 in PBS). Serum samples, controls, and calibrators were diluted fresh at 1 : 320 using a solution containing 0.1% BSA in PBS supplemented with 0.1% nonimmune donkey serum and 0.1% Tween-20. The calibration curve was prepared by serial dilution at a factor of 10 of a commercial chimeric mouse scFv fused with human IgG1 Fc anti-SARS-CoV-2-S1-RBD (CSB-YP3324GMY1, Cusabio). 100 *μ*L of each dilution was seeded in duplicate and incubated for 1 hour at room temperature (20 ± 2°C). Then, all liquids were removed and the plate rinsed 5 times with 250 *μ*L of washing solution with 2 sec shaking and 10 sec soaking each time in an automatic microplate washer.

Later, each well was incubated at 20°C for 1 h with 100 *μ*L of 20 ng/mL solution of peroxidase-conjugated AffiniPure Donkey Anti-Human IgG, Fc*γ* fragment specific (709-035-098, Jackson ImmunoResearch). Finally, the assay was developed using 3,3′,5,5′-tetramethyl-benzidine substrate (T0440, Sigma-Aldrich) and stopped after 15 minutes with 2 M sulfuric acid; then, the absorbance was measured at 450 nm in a Cytation 5® plate reader (BioTek). For interpretation, we have the following: (1) qualitative absorbance ratio between the unknown sample and a calibrator (positive ratio > 1.1;negative ratio < 0.9; undetermined1.1 < ratio > 0.9) and (2) quantitative interpolation from a standard calibration curve. Blank, controls, calibrators, and curves were performed within the respective 5-10% coefficient variation (CV).

Sera previously titered using an IVD ELISA test (Euroimmun) were used as internal positive controls (ratio above 2.5, 7.5%CV), as a calibrator (ratio 1.0 ± 0.05, <5%CV), and as internal negative controls (ratio below 0.8, 5%CV). Additionally, a calibration curve between 0.0 and 250.0 ng/mL of specific IgG was used, and it included as a reference material a commercial chimeric mouse scFv fused with human IgG1 Fc anti-SARS-CoV-2-S1-RBD (CSB-YP3324GMY1, Cusabio).

#### 2.2.1. Neutralization Assay

Samples were diluted in DMEM, and 50 *μ*L of dilution was added to each well in triplicate. Three to 5 pg of a luciferase-coding SARS-CoV-2 pseudotype was prepared freshly, mixed 1 : 1 with a diluted sample, and incubated for 1 hour at 37°C. DMEM alone was used as a positive control (100% infectivity). Then, 1 × 10^4^ of HEK-ACE2 cells were incubated with each sample mix and cultured for 48 h before measuring the firefly-luciferase activity. Neutralizing assays were validated considering the following pass/fail criteria: (1) The average relative light units (RLU) of the pseudovirus control wells are ≥10 times the average RLU of the negative control wells (HEK293T cells). (2) The coefficient of variation (CV) between RLU in the pseudotype control wells is ≤30%. (3) The percentage difference for triplicate wells is ≤30% for sample dilutions that yield at least 40% neutralization. (4) Positive control neutralization curve crosses the 50% neutralization cut-off 0-1 times. (5) Finally, sigmoid curves and estimation of infectious dose 80 (ID80) were obtained using a 4-parameter nonlinear regression curve fit [19].

### 2.3. Statistical Analyses

Statistical analysis was performed using GraphPad Prism software, version 8.0. Qualitative and quantitative correlation was assessed using Pearson's correlation coefficient. Fisher's contingency test was used to assess the IgG levels and neutralization activity. Differences in mean values between groups were analyzed by the Mann–Whitney test. All values were depicted as a geometric mean with 95% CI. One-way ANOVA and Tukey's multiple comparison test were performed for statistical analysis between various variables. The critical value for statistical significance was established as *p* ≤ 0.05. Values marked with asterisks mean the following: ^∗^*p* < 0.05, ^∗∗^*p* < 0.01, ^∗∗∗^*p* < 0.001, and ^∗∗∗∗^*p* < 0.0001.

## 3. Results

### 3.1. Objective Metrics for Stratification and Plasma Donor Selection Based on Anti-RBD IgG Levels

For this research, we developed and validated a qualitative ELISA for the detection of a human IgG anti-S1-RBD fragment of SARS-CoV-2 (anti-RBD IgG). Previously, a qualitative ELISA from our laboratory showed 99% sensitivity and 85% specificity, and a percentage of agreement of 92.1 compared to the IVD ELISA from Euroimmun (Cereceda et al., 2020, submitted). For all our analyses, each plate used was coated with 50 ng of antigen in a proportion of 1.8 pmol of S1-RBD fragment per cubic centimeter of active surface. Using a commercial chimeric mouse scFv fused with human IgG1 Fc anti-SARS-CoV-2-S1-RBD (CSB-YP3324GMY1, Cusabio) as reference material, our improved ELISA showed a linearity between 6.25 and 50 *μ*g/mL, a limit of detection (LoD) of 6 ng/mL, and 40 ng/mL as limit of quantification (LoQ) for the specific IgG. This assay showed for the qualitative and quantitative analyses a direct proportionality and correlation with Pearson's *r* coefficient of 0.9916 with a 95% CI from 0.9523 to 0.9985 and a*p*value < 0.0001, allowing both metrics for sample analysis (Supplemental Figure S1). Using 100 samples previously tested for anti-SARS-CoV-2 IgG, the assay showed 99% specificity and 98.5% sensitivity, and a correlation of 92%. Based on the covariance observed in a local group never exposed to SARS-CoV-2 (mean 2.4 *μ*g/mL), the cut-off for positivity was estimated using the mean plus 3 standard deviation (mean + 3SD) to consider 99.73% of the negative population in a normal distribution (Supplemental Figure S1), and the calculated value was 6.6 *μ*g/mL of anti-RBD IgG in serum.

### 3.2. Anti-RBD IgG Seroconversion in Individuals Exposed to SARS-CoV-2 in Santiago, Chile

**Figure 1:**
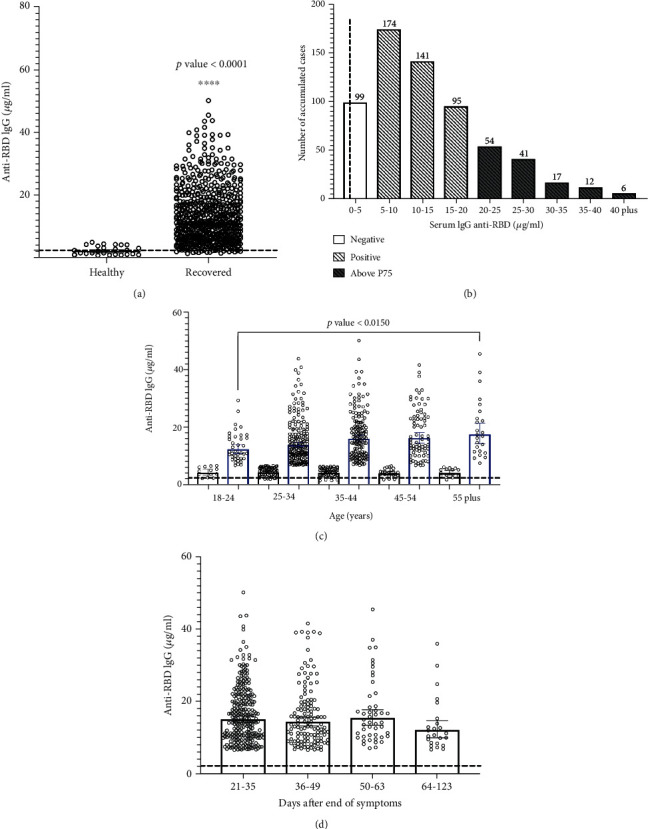
Analysis of anti-RBD IgG in individuals exposed to SARS-CoV-2 in the metropolitan region of Chile. (a) Anti-RBD IgG levels in participants, namely, healthy controls as the baseline group (*N* = 25), and participants who recovered from COVID-19 with different days of convalescence (*N* = 639). (b) Histogram for accumulated frequency and antibody concentration range. Positive and negative ELISA results are indicated. Darker bars represent the 75th percentile (P75) for the studied cohort. (c) Anti-RBD levels based on age for positive (blue bars) and negative (black bars) cases. (d) Levels of IgG after symptom offset in the convalescent group and positive qualitative ELISA. (a–d) In all cases, the dashed line corresponds to the healthy controls.

**Figure 2:**
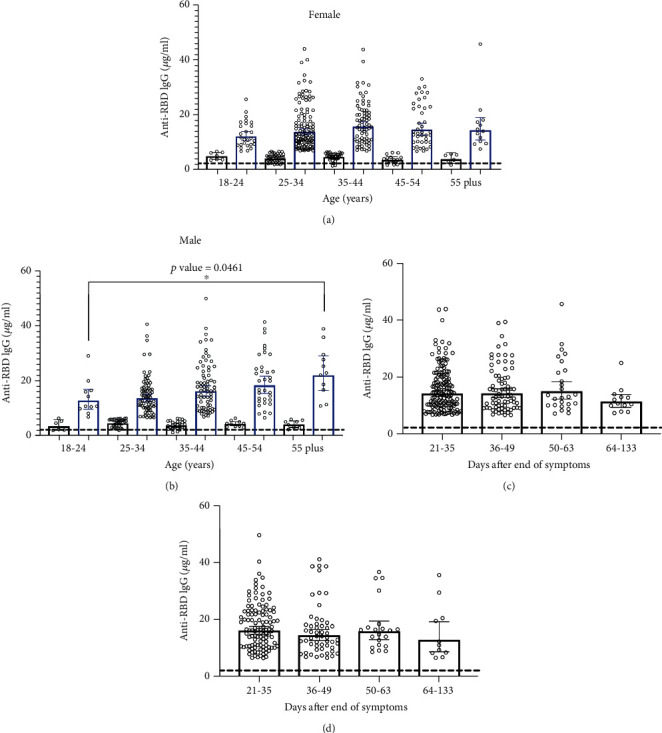
Stratified anti-RBD IgG levels by gender, age, and days of convalescence. Measured IgG distribution for positive (blue) and negative (black) quantitative ELISA. Cases were segregated as female (*N* = 361) (a, c) and male (*N* = 278) (b, d) based on age and days after symptom offset. In all cases, the dashed line corresponds to the control group.

639 serum samples were collected in Santiago, Chile, during July and August 2020, and were analyzed to estimate the seroconversion and quantification of specific anti-RBD IgG. This cohort included convalescent cases between 21 and 123 days after their symptoms were offset, with a median of 34 days (Supplemental Table 1). All patients recovered from mild to moderate COVID-19 without special medical requirements. Overall, the infection with SARS-CoV-2 promoted the specific production of antibodies in infected people, and their concentrations in blood were substantially and statistically higher than asymptomatic never-exposed people (*p* < 0.0001) (Figure 1(a)). In fact, the segregation of positive and negative cases using a quantitative cut-off showed a positivity of 72.9% (466 of 639), with a geometric mean of 14.84 *μ*g/mL and a calculated 75th percentile (P75) of 20.9 *μ*g/mL of anti-RBD IgG in serum for that group; most of the positive cases showed a range between 5 and 20 *μ*g/mL (Figure 1(b)). The convalescent population included in this study had a median age of 35 years old; when the levels of IgG were analyzed by participant's age, they showed a high dispersion with no significant differences between groups, except between the two extreme age groups (18-24 versus 55-plus years old) where higher levels of anti-RBD were observed for older people (*p* = 0.015) (Figure 1(c)). Regarding the time after symptoms were offset, the global average concentration of anti-RBD IgG did not show any statistical differences between groups (Figure 1(d)). When the population was segregated by gender, no statistical differences were observed (Figures 2(a) and 2(b)); however, a trend of higher levels of specific IgG were observed for older men compared with the younger group (*p* = 0.0461) (Figure 2(b)). The amounts of anti-RBD antibodies were not influenced by the time after symptoms ended and did not show any statistical differences between men or women groups (Figures 2(c) and 2(d)).

Regarding the 75th percentile (P75) by gender, men showed statistically higher levels of IgG than women when P75 was estimated for each independent gender group (high male vs. high female, *p* = 0.0030), and specifically 67 of 268 women (25%) and 48 of 198 men (24.2%) were above the global P75 with an average concentration of specific IgG of 26.5 *μ*g/mL and 29.6 *μ*g/mL, respectively (Supplemental Figure S2).

### 3.3. Anti-RBD IgG Kinetic and Neutralization Activity in Convalescent Plasma

**Figure 3:**
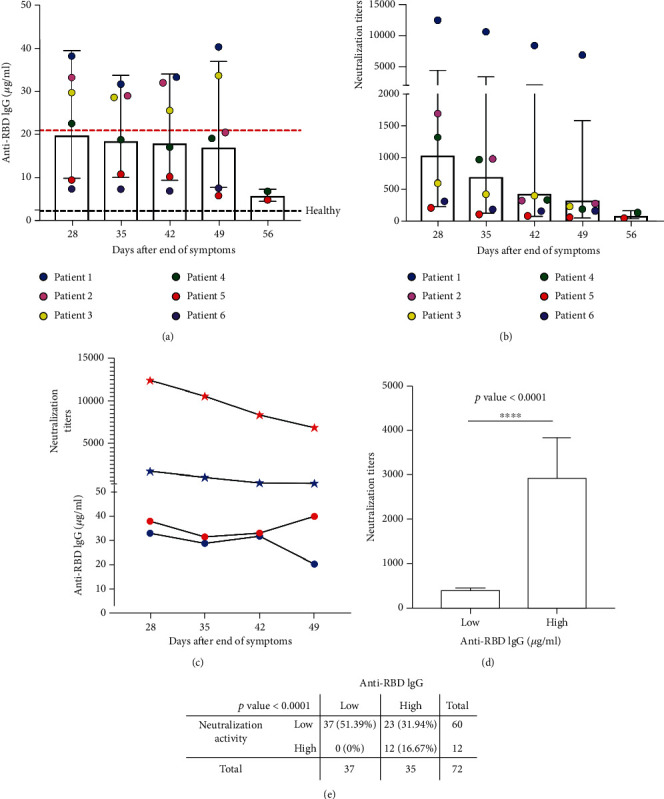
Convalescent plasma donors and IgG antiviral levels. Anti-RBD IgG concentration (a) and neutralizing activity (b) were determined at different days of convalescence. In (c), representative cases with a similar IgG concentration and decreasing neutralizing antibodies. (d) Neutralization activity and IgG levels; average neutralizing activity was represented for stratified high (*N* = 20) and low (*N* = 52) IgG anti-RBD cases based on the 75th percentile. The results are presented as the mean ± SEM, and statistical differences from the Mann–Whitney test are shown. (e) 2×2 contingence analysis for high/low antibody levels (P75) and high/low neutralizing activity (median ID80). 75th percentile (P75) and IgG cut-off are indicated in red and black dotted lines, respectively.

To evaluate the persistence of antibodies against SARS-CoV-2 after infection, six cases were under follow-up and evaluated for up to 50 days of convalescence, and the blood collection started 28 days after their symptoms finished. In addition, the neutralization activity in serum was measured as the 80% Inhibitory Dilution (ID) using pseudoviral particles [19]. The cases showed anti-RBD IgG levels below and above the measured P75 (concentration = 20.9 *μ*g/mL) but significantly higher than the control group, and a broad range of neutralizing ID80 between 83 and 12,400 (Figures 3(a) and 3(b)). Individuals with a similar IgG concentration exhibited dramatic differences in the ability to neutralize the virus *in vitro*, up to 100-fold higher than others, and the starting amount of IgG did not reflect the observed decaying curve for the specific IgG or neutralizing activity (Figures 3(b) and 3(c)). Overall, the quantified concentration of anti-RBD IgG correlated with the presence of SARS-CoV-2 NAb (Pearson's *r* = 0.51, *p* = 0.001) (data not shown). Further analysis considering 72 unrelated cases showed that serum samples with antibodies below P75 had statistically very low levels of neutralization (*p* < 0.0001) (Figure 3(d)) and, when the patients were selected based on IgG above that cut-off, those showed a significantly increased proportion of NAb (*p* < 0.0001) (Figure 3(e)).

### 3.4. Early Seroconversion in Individuals with an Active Infection of SARS-CoV-2

**Figure 4:**
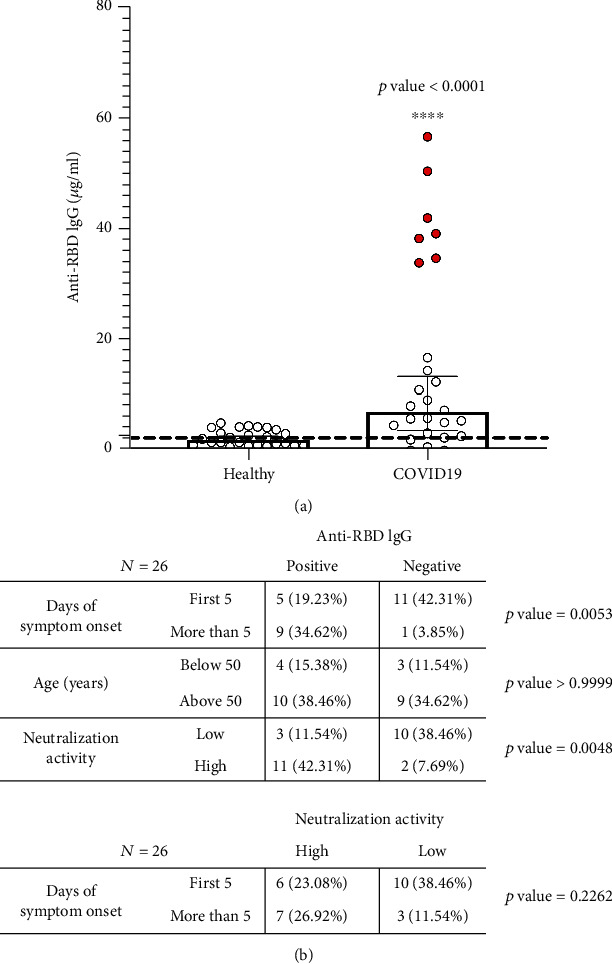
Anti-RBD IgG antibodies and neutralization activity during active infection with SARS-CoV-2. (a) Anti-RBD IgG levels in participants, namely, healthy controls as the baseline group (*N* = 25) and active COVID-19 patients with different degrees of severity (*N* = 26). (b) 2 × 2 contingence analysis for positive/negative antibody levels and days of symptom onset, age (years), neutralization activity and for high/low neutralization activity (median ID80) and days of symptom onset. 75th percentile (P75) is indicated in red circles, and IgG cut-off is indicated by a black dashed line.

We analyzed samples from 26 patients who were categorized as severe COVID-19 cases within the first 14 days of their diagnosis of an infection with SARS-CoV-2 (Table 1). This group showed 53.8% positivity (14 of 26) with a mean concentration of 21.2 *μ*g/mL of anti-RBD IgG, and 7 out of the 26 patients (27%) had antibodies above the 75th percentile (20.9 *μ*g/mL) (Figure 4(a), red circle). We did not observe correlations in IgG levels and previous medication to the blood drawing (Table S2), and only one case received hydroxychloroquine as treatment of a systemic lupus erythematosus before the COVID-19 diagnosis. A contingency analysis showed a correlation between days of disease and seroconversion (*p* = 0.0053), increasing the frequency of positivity after 5 or more days after the symptoms started. However, 5 of 26 cases (19.2%) developed antibodies against RBD within the first 5 days (Figure 4(b)). No correlation between age at diagnosis and early seroconversion was observed (*p* > 0.9999) (Figure 4(b)). As we expected, the presence of anti-RBD antibodies correlated with the ability to neutralize SARS-CoV-2 in vitro (*p* = 0.0048), but the magnitude of the measured neutralizing activity in serum from patients with a severe COVID-19 was independent of the days after symptoms started (*p* = 0.2262) (Figure 4(b)).

## 4. Discussion

Early results from the Hubei province of China showed a polyclonal IgG prevalence against SARS-CoV-2 of 89.8% (95% CI 88.2-91.3%) in 1,740 COVID-19 convalescents [20], and the severity of the symptoms during COVID-19 have been related to the duration of humoral response, where asymptomatics or individuals with a mild illness have shown a quicker reduction in antibody titers than more severe cases [21, 22]. In the current study, 50% of the convalescent population was considered within the range between 18 and 68 years old with a median age of 35, which represents the main workforce in Chile. The estimated seroconversion was 72.9%, which did not show significant differences at the level of gender, age, and days after symptom offset; however, the serum concentration of IgG anti-SARS-CoV-2 varied dramatically between cases, and it was not reflected in the measured neutralizing activity. Our observations were consistent with the previously reported seroconversion rates after four weeks of symptoms [20, 22, 23]. It is interesting that the Chilean convalescent population of this study showed similar results compared to a group of 49 recovered COVID-19 patients recruited in Wuhan, China, between February and March 2020 [24]. In 2020, Li et al. reported that 28 days after the onset of symptoms, 90% of the group (18-55 years old) showed an S-RBD-specific IgG titer above 1 : 160 and 78% had a titer of 1 : 640 or higher [24], which means that 90% of that group seroconverted after infection, and the S-RBD-specific and N-specific IgG antibodies increased after 4 weeks from the onset of symptoms, with no significant correlation to age, sex, or ABO blood type [24]. It is important to consider that an IgG response against the spike RBD domain has been associated with a patient's improved survival independently of other factors such as sex or age, supporting the concept that these antibodies are a significant contributor to the protective effect of humoral immunity in COVID-19 [25]. Additionally, we observed a significantly higher amount of anti-RBD IgG in older men, and it was previously shown in a preprint article where the authors suggested that being male, an older adult, and being hospitalized with COVID-19 were each associated with having greater neutralizing antibody titers and IgGs against the S1-domain, the S1-RBD fragment, or the full spike protein [26].

Several authors have reported a rapid seroconversion during the first weeks of symptoms [23, 25, 27]; we observed 53.8% of IgG seroconversion in severe COVID-19 patients between 4 and 9 days after a positive PCR test. A very early study reported for a small group (17) showed an anti-RBD-specific seroconversion rate of 64.71% for IgG within the 4-10 days after illness onset; however, after 15 days of disease, the seroconversion rate reached 100% (169 cases) for the three immunoglobulins [28]. Another study which considered 28 mild and 7 severe COVID-19 cases showed that IgG in serum was detected in 1 of the 35 cases at the first week after the date of illness onset; however, at the third week, 68.9% (*n* = 20/29) of the patients seroconverted in mild cases, while it was 100% (*n* = 7/7) in severe cases [29]. Recently, it was reported from 166 studied cases that patients within the first week of symptoms with a very severe case of COVID-19 (*n* = 45) had a quicker and more significant anti-RBD-specific seroconversion than hospitalized patients without ICU requirements (*n* = 35) or outpatients with mild to moderate symptoms (*n* = 86). Curiously, at the second week, all the inpatient cases had statistically higher levels of the specific IgG than the outpatient group [22], which was consistent with another report [30].

Typical antibody responses to acute viral infection are quickly induced in COVID-19 patients as shown by Brouwer et al. in 2020, where the seroconversion rate and antibody levels increase rapidly during the first two weeks and the cumulative seropositive rate reaches 50% on the 11th day and 100% on the 39th day [25]. As other authors reported, antibodies against the RBD of spike can be detected at the median of 11 days after the onset of symptoms and the timing of seroconversion may not correlate with the clinical course [6, 8].

In 2020, Gozalbo-Rovira showed an anti-RBD IgG threshold above 1.15 AU/mL as a predictor of neutralizing antibody (NAb) titers, suggesting that quantitative IgG levels may correlate with neutralizing titers with high sensitivity and specificity [27]. In general, more than 70% of the population included in this study developed anti-RBD IgG antibodies, and 24.9% of the positive cases were above 20.9 *μ*g/mL, suggesting a heterogeneous seroconversion but a sustained production of the specific IgG up to 100 days after symptom offset, independenly of age and gender. All convalescent participants showed neutralizing activity with a variability up to 100 times between cases. However, high levels of specific IgG, even though above P75, did not guarantee a significant antiviral or neutralizing activity in plasma. These results were concordant with the results shown by Wang in 2020, where IgG levels (anti-S or anti-N) exhibited a moderate correlation with neutralization titers in plasma (Pearson's *r* = 0.5393 and *r* = 0.6709, respectively) [32]. A theoretical modeling report suggested that the probability of detecting IgG could reach a maximum of around 25-27 days after COVID-19 symptoms end, with a predicted positivity between 98% and 100% of individuals for anti-SARS-CoV-2 IgG [26]. The report also predicted that the probability of detecting NAb in plasma may rapidly rise to near 100% around 29 days after symptoms end without significant differences between mild/moderate and severe/clinical cases [33]. Previously, a trial using COVID-19 convalescent plasma was halted prematurely because 80% (53 of 66) of symptomatic participants for only 10 days at the time of enrolment showed seroconversion [27]. In addition, the authors reported SARS-CoV-2 neutralizing activity in 79% of the plasma receptors before transfusion (44 of 56) with median titers comparable to the donors (1 : 160 vs. 1 : 160, *p* = 0.40). Our results have confirmed that most of the Chilean population which recovered from COVID-19 developed anti-SARS-CoV-2 antibodies even only a few days after infection as shown in patients with critical conditions. The quantification of anti-RBD IgG within the local population is necessary as an objective criterion to estimate the neutralizing activity as a protective biomarker within the infected population with SARS-CoV-2. It may be also considered when the laboratory measurement of neutralizing antibodies is limited. As a limitation of this study, the reduced number of cases for some analysis do not allow us to consider our cohort as representative of a larger population. We did not include cases with active disease classified as mild or moderate COVID-19, so we cannot estimate if there is or is not an association between symptomatology and early seroconversion.

## 5. Conclusions

In general, more than 70% of the population included in this study developed anti-RBD antibodies with a variable neutralizing activity, suggesting a heterogeneous seroconversion after recovering from COVID-19 with a sustained production of specific IgG up to 100 days, independent of age and gender.

## Data Availability

The clinical data used to support the findings of this study are available from the corresponding authors upon request.

## Supplementary Materials

Supplementary Materials Supplementary Figure 1: qualitative and quantitative ELISA performance. In (a), a histogram is shown with the calculated absorbance ratio in a qualitative analysis (positive, ratio above 1.1 as dotted line) and its corresponding anti-RBD IgG concentration range. Data are shown as mean ± 95 C.I. (b) Correlation between qualitative and quantitative ELISA analysis; average values for absorbance ratio and concentration are represented as continuous variables. Supplementary Figure 2: high and low levels of anti-RBD IgG in recovered COVID-19 patients. High (*n* = 116) and low (*n* = 350) levels were estimated as above or below the 75th percentile for the global cohort (a) or separated by gender (b). In both cases, the dashed line corresponds to the group of healthy individuals. Asterisks indicate significant differences between groups and analyzed with the Mann–Whitney test. ^∗∗∗∗^*p* < 0.0001.
