# Supplementary material for: Seroconversion and Abundance of IgG Antibodies against S1-RBD of SARS-CoV-2 and Neutralizing Activity in the Chilean Population
Source: J Immunol Res. 2021 Feb 19;2021:6680337. doi: 10.1155/2021/6680337 (PMC7901042; doi:10.1155/2021/6680337)
Supplement: Supplementary Materials — Supplementary Figure 1: qualitative and quantitative ELISA performance. In (a), a histogram is shown with the calculated absorbance ratio in a qualitative analysis (positive, ratio above 1.1 as dotted line) and its corresponding anti-RBD IgG concentration range. Data are shown as mean ± 95 C.I. (b) Correlation between qualitative and quantitative ELISA analysis; average values for absorbance ratio and concentration are represented as continuous variables. Supplementary Figure 2: high and low levels of anti-RBD IgG in recovered COVID-19 patients. High (n = 116) and low (n = 350) levels were estimated as above or below the 75th percentile for the global cohort (a) or separated by gender (b). In both cases, the dashed line corresponds to the group of healthy individuals. Asterisks indicate significant differences between groups and analyzed with the Mann–Whitney test. ∗∗∗∗p < 0.0001. [file 6680337.f1.pdf]

SUPPLEMENTARY DATA

"SEROCONVERSION AND ABUNDANCE OF IgG ANTIBODIES AGAINTS S1-RBD OF SARS-COV-2 AND NEUTRALIZING ACTIVITY IN CHILEAN POPULATION"

González-Stegmaier, R., Cereceda, K., Briones, JL., Beltran-Pávez, C., Oyarzún-Arrau, A., Riquelme-Barrios, S., Selman, C., Yarad, F., Mahave, M., Caglevic, C., Morales, R., Aguirre, A., Valiente-Echeverría, F., Soto-Rifo, R., Marsiglia, H., Gazitua, R., and Villarroel-Espíndola, F.

SUPPLEMENTARY TABLE S1

	**Convalescent**	**Active**	**Healthy**
Total cases	690	26	25
Age Range (years)	18-68	19-83	28-53
Women	361	9	7
Men	278	17	18
Sample collection			
Days after symptoms onset	NA	0-14 (3.5±3.8)*	NA
Days after symptoms offset	21-123 (34±12.7)*	NA	NA
Symptomatology during COVID-19	Mild to Moderate	Severe	NA
Symptomatology at collection	Asymptomatic to Mild	Critically ill	Asymtomatic
PCR test	Positive	Positive	Negative

*median±SD

SUPPLEMENTARY TABLE S2

Active COVID cases	**N=26**
mean age (years)	54.76
Treatments previous to the blood collection	**N**
Hydroxychloroquine	1
Chloroquine	0
Azithromycin	11
Lopinavir/Ritonavir	0
Tocilizumab	0
Remdisivir	0
ACE inhibitors	0
Angiotensin II receptor blockers	0
Methylprednisolone	4
Betamethasona	0
Dexamethasone	1
Prednisone	0
Hydrocortisone	1
None	8

SUPPLEMENTARY TABLE S3

	Qualitative ELISA	Quantitative ELISA
S1-RBD antigen bound	50 ng	50 ng
Sample dilution	1:320	1:320
Sample Incubation time	1 hour	1 hour
HRP-conjugated antibody	20 ng/ml	20 ng/ml
conjugated incubation time	1 hour	1 hour
Developing time	15 min	15 min
Principle method	Qualitative absorbance ratio between the unknown sample and a calibrator	Quantitative interpolation from a standard calibration curve
Calibrator	Known standard serum	not requiered
Positive Control	Titered known serum (absorbance ratio >2.5, 7.5%CV)	Known positive serum (absorbance 1.63±0.08, 5%CV)
Negative Control	Titered known serum (absorbance ratio <0.8, 5%CV)	Known negative serum (absorbance 0.26±0.01, 5%CV)
Blank	not required	Sample dilution buffer
Calibration curve	not required	human IgG1 anti-S1-RBD
Linearity	Not applicable	6.25 - 50.00 µg/mL
Interpretation	Positive: ratio>1.1; Negative: ratio<0.9; Undeterminated: 1.1-0.9	Positive: calculated value above the cutoff (healthy population)
Sensitivity	99.0%	99.0%
Specificity	85.0%	98.5%

SUPPLEMENTARY FIGURES

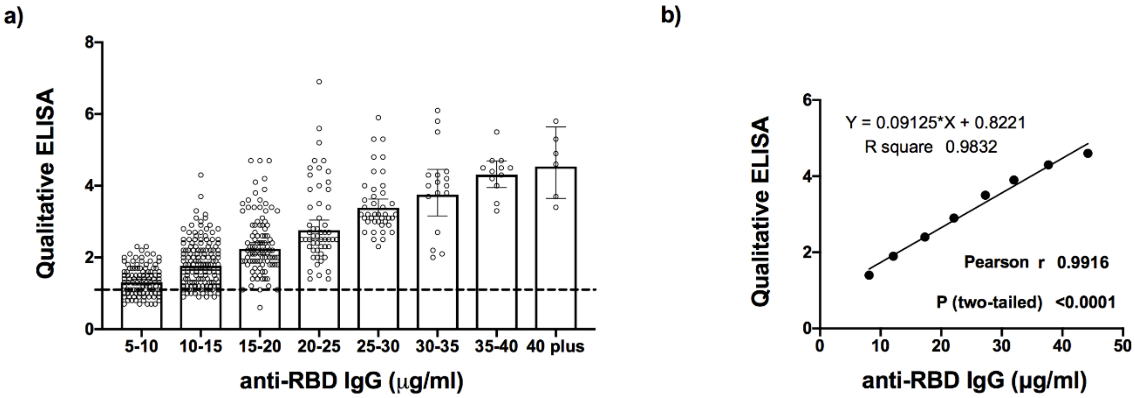

Supplementary Figure 1: Qualitative and quantitative ELISA performance. In a) Histogram with the calculated absorbance ratio in a qualitative analysis (positive, ratio above 1.1 as dotted line) and its corresponding anti-RBD IgG concentration range. Data were shown as mean±95IC; b) correlation between qualitative and quantitative ELISA analysis, average for absorbance ratio and concentration were represented as continuous variables.

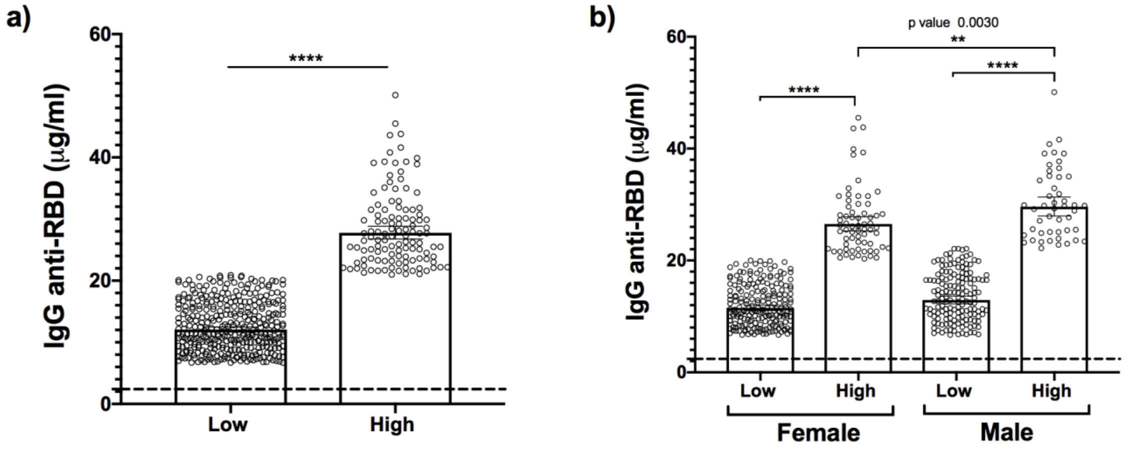

Supplementary Figure 2: high and low levels of anti-RBD IgG in recovered COVID-19 patients. High (n=116) and low (n=350) levels were estimated as above or below the 75th percentile for global cohort (a) or separated by gender (b). In both cases the dotted line corresponds to the group of healthy individuals. Asterisks indicate significant differences between group and analyzed with Mann-Whitney test. ****p<0.0001.
